# Intestinal Stem Cells to Advance Drug Development, Precision, and Regenerative Medicine: A Paradigm Shift in Translational Research

**DOI:** 10.1208/s12248-017-0178-1

**Published:** 2017-12-12

**Authors:** Jonathan P. Mochel, Albert E. Jergens, Dawn Kingsbury, Hyun Jung Kim, Martín G. Martín, Karin Allenspach

**Affiliations:** 1 Department of Biomedical Sciences, Iowa State University College of Veterinary Medicine, 2448 Lloyd, 1809 S Riverside Dr., Ames, Iowa, 50011-1250, USA; 2 Department of Veterinary Clinical Sciences, Iowa State University College of Veterinary Medicine, 50011-1250 Ames, Iowa, USA; 3 Department of Biomedical Engineering, University of Texas at Austin, Austin, Texas 78712, USA; 4 Department of Pediatrics, University of California Los Angeles, California, Los Angeles 90095-1782, USA

**Keywords:** dog, enteropathies, organoid, precision medicine, transplantation

## Abstract

Recent advances in our understanding of the intestinal stem cell niche and the role of key signaling pathways on cell growth and maintenance have allowed the development of fully differentiated epithelial cells in 3D organoids. Stem cell-derived organoids carry significant levels of proteins that are natively expressed in the gut and have important roles in drug transport and metabolism. They are, therefore, particularly relevant to study the gastrointestinal (GI) absorption of oral medications. In addition, organoids have the potential to serve as a robust preclinical model for demonstrating the effectiveness of new drugs more rapidly, with more certainty, and at lower costs compared with live animal studies. Importantly, because they are derived from individuals with different genotypes, environmental risk factors and drug sensitivity profiles, organoids are a highly relevant screening system for personalized therapy in both human and veterinary medicine. Lastly, and in the context of patient-specific congenital diseases, orthotopic transplantation of engineered organoids could repair and/or replace damaged epithelial tissues reported in various GI diseases, such as inflammatory bowel disease, cystic fibrosis, and tuft enteropathy. Ongoing translational research on organoids derived from dogs with naturally occurring digestive disorders has the potential to improve the predictability of preclinical models used for optimizing the therapeutic management of severe chronic enteropathies in human patients.

## INTRODUCTION

**Fig. 1:**
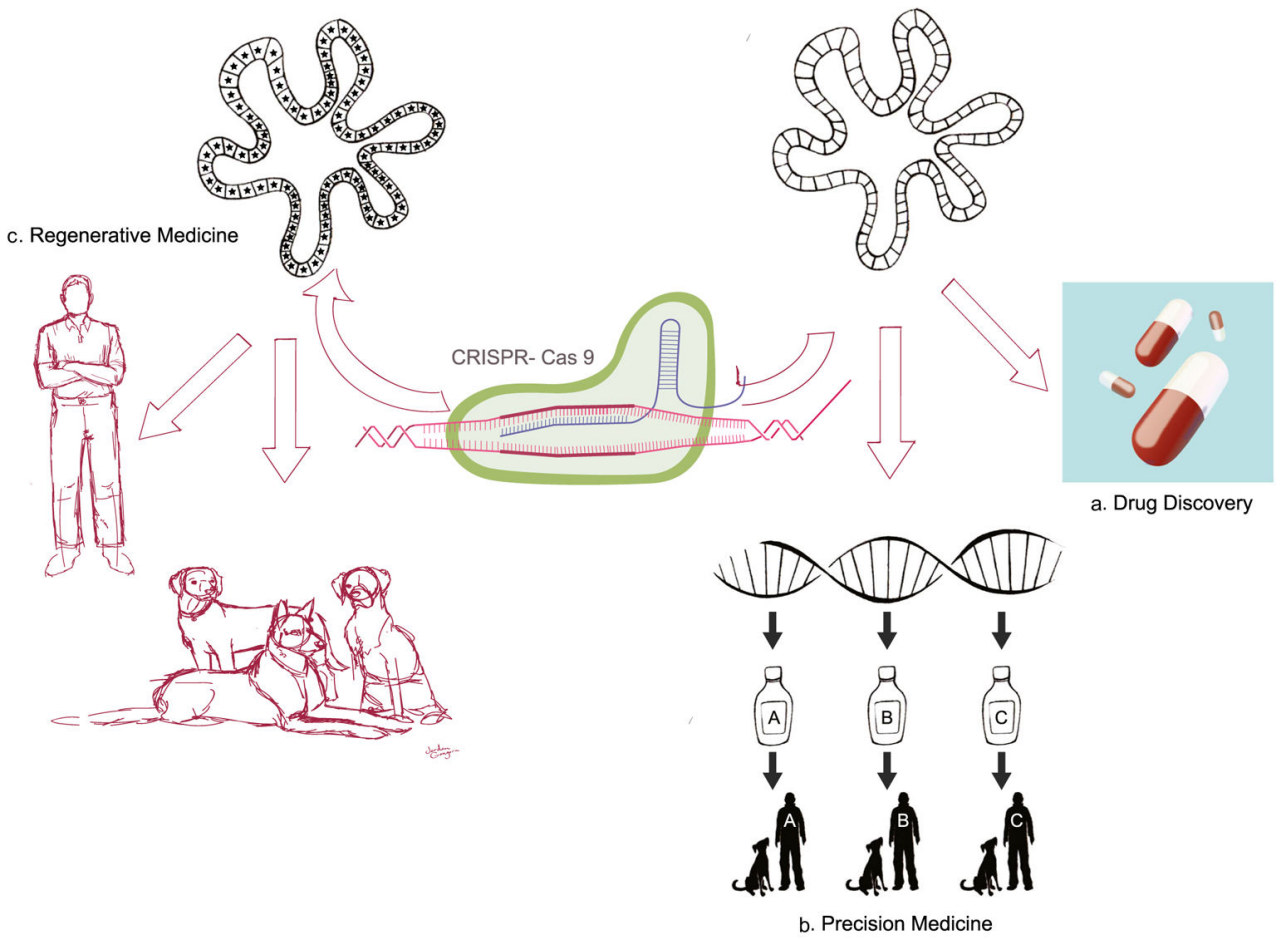
Organoids: a promising *in vitro* system for drug discovery, precision, and regenerative medicine in human and veterinary medicine. (A) Organoids can be used as a preclinical model to evaluate the efficacy and safety of candidate drugs prior to live studies in animals and humans. (B) In addition, because they are derived from individuals with different genotypes, organoids are a relevant screening system for precision medicine. (C) Finally, transplantation of genetically engineered organoids has the potential to repair and/or replace damaged epithelial tissues associated with several gastrointestinal diseases

Recent advances in biomedical research have allowed the development of intestinal stem cells in three-dimensional (3D) culture systems supporting *ex vivo* epithelial growth into organoids [1, 2]. Stem cell-derived organoids have multiple advantages over traditional 2D epithelial systems utilizing cancer-derived cell lines (*e.g.*, Caco-2, T84, and HT29) [3], or spontaneously immortalized epithelial cells (*e.g.*, rat intestinal epithelial (RIE) cultures) which rarely reproduce the structure and function of the intestinal epithelium. The benefit of the 3D organoid culture lies in the method’s ability to better harness innate endogeneous cellular programming within higher order cellular tissue organization [4]. The development of an *ex vivo* gut microphysiological system that morphologically, biologically, and chemically replicates the endogenous epithelium shows tremendous potential to study the biology of epithelial diseases [5, 6], and to evaluate the efficacy and toxicity of orally administered therapeutic drugs (Fig. 1). Furthermore, organoids may be collected from hosts having different genotypes, environmental risk factors (*e.g.*, diet, microbiota), or drug sensitivity profiles, thereby more faithfully reflecting the diversity of the host background when cultured *ex vivo*. Yet, more data are needed to link the information derived from the use of organoids to the pathogenesis of the diseases of interest before it can be used as a primary tool to optimize individualized therapeutic options within a clinical setting.

The present Commentary provides a review of the current knowledge on the biology of intestinal organoids, their potential value in drug discover, precision medicine and regenerative (*i.e.*, transplantation medicine), and the remaining gaps that need to be resolved before it can be used in a patient-specific manner.

## DEFINITIONS, BACKGROUND, AND RATIONALE

### Organoids: What they Are and why they Are Relevant to Study the Biology of Intestinal Diseases?

Organoids can be propagated from embryonic, induced pluripotent, or leucine-rich repeat containing G protein-coupled receptor 5 (Lgr5)-positive stem cells, also known as crypt base columnar (CBC) cells, located in the intestinal crypt. Primary cultures developed from CBC cells or isolated intestinal crypts are termed “enteroids” or “colonoids,” depending on the anatomic region (*i.e.*, small *vs*. large intestine) they are derived from [7]. Additionally, 3D cancer cell cultures can be obtained from individual biopsies of patients and are referred to as “tumor organoids.” For simplification purposes, the generic term “organoid” will be used consistently throughout the rest of the Commentary.

A rich body of literature has shown that 3D cell cultures are superior to traditional 2D monolayer systems in mimicking complex *in vivo* cellular heterogeneity [8–10]. The development of 3D organoid models comprised of multiple cell types provides an attractive approach to investigate key intra- and intercellular signaling pathways that contribute to the development of chronic enteropathies. Specifically, intestinal organoids can facilitate detailed mechanistic studies on the molecular and cellular reprogramming events that occur during inflammatory bowel disease (IBD) and colorectal cancer (CRC) pathogenesis. As described by Thoma *et al.* [11], multiple levels of complexity can be built upon in 3D culture systems, ranging from simpler models of cancer cell monocultures in liquid-based environments to more advanced models that include co-cultures with endothelial, bacterial [12], and immune cells. These more complex systems can also be used to study the effect of environmental chemicals (such as endocrine disruptors) on disease development [13]. The use of organoids as an enteric infection model for bacteria and viruses crystallizes another recent application of 3D models for understanding disease pathophysiology. This was illustrated in a study by Rouch *et al.* [14] where the authors developed a human organoid model to elucidate the role of intestinal microfold (M) cells on the proliferation of *Salmonella typhimurium*.

### Comparison to Caco-2 Cells for the Study of Oral Drug Transport and Metabolism

The Caco-2 cell model derived from human colon adenocarcinoma primarily measures passive transcellular and paracellular permeability of test compounds. Compared to *in vivo* physiology, solute carrier transporters (*e.g.*, organic anion and cation (OAT/OCT) transporters) in the Caco-2 system are known to be expressed in relatively low amounts, such that the permeability of oral drugs is typically underestimated in this model [15]. This has been demonstrated for β-lactam antibiotics (*e.g.*, cephalexin and amoxicillin), which are completely absorbed *in vivo* despite being poorly permeable across the Caco-2 cell monolayer [16, 17]. Overall, while the Caco-2 model can be considered relevant for predicting the oral availability of highly permeable drugs, it usually performs poorly in accurately predicting the gastrointestinal (GI) absorption of low permeability compounds [15]. In addition, while human-derived Caco-2 cells are a commonly used *in vitro* system to study P-glycoprotein (P-gp)-mediated drug efflux, there are several limitations to this model, including the presence of tighter cellular junctions than observed in normal enterocytes. Using 3D organoids, Zhao *et al.* [8] were able to study P-gp-mediated efflux in the presence of Rhodamine 123 which they later established as a predictive model for P-gp inhibitor drug screening in humans. Finally, although Caco-2 cell-based models are known to express hydrolase, esterase, and brush-border enzymes, they typically fail to express appreciable amounts of CYP3A4, one of the main cytochromes contributing to the pre-systemic loss of many orally administered drugs [13]. Negoro *et al.* [9] have successfully established an organoid model that proved to be relevant in the evaluation of drug-mediated CYP3A4 induction on victim drug oral bioavailability and could be used as an alternative to the Caco-2 system.

### Organoids and Organs-on-a-Chip

Organs-on-a-chip (OAC) are a recently emerged microphysiological system that leverages computer microchip manufacturing technology to create a microfluidic cell culture device [18]. OAC aims to emulate organ-specific physiological functions or cellular responses in a defined 3D microarchitecture and dynamic biomechanical motions of the target organ. Typically, microfluidic OAC contain multiple juxtaposed microchannels compartmentalized by a flexible or a rigid porous membrane to reform a tissue-tissue interface. This organ-specific microenvironment allows inter-cellular interactions, for instance of the human intestine, between the epithelium, endothelium, mesenchymal cells, immune components, and living gut bacteria [19]. More importantly, physiologically relevant cultures in the OAC enable to reprogram cell morphogenesis, differentiation, and cellular responses to mimic the complex *in vivo* environment. For example, when Caco-2 cells are cultured in the gut-on-a-chip, cells spontaneously undergo villus morphogenesis with crypt-villus characteristics, differentiate into four lineages of human small intestinal cells (*i.e.*, absorptive, goblet, enteroendocrine, and Paneth cells), and display key physiological functions such as enhanced barrier function, mucus production, and CYP3A4-mediated drug metabolism [20]. is notable that conventional 2D static culture of Caco-2 cells never reproduces these reprogrammed functions *in vitro*. Such features can help deciphering the pathophysiology of chronic human enteropathies such as IBD, CRC, ileus, or celiac disease.

Despite the great promise of the OAC technology, the transfer of cells from a macroscopic environment (*e.g.*, dishes, flasks, and well-plates) to a microfluidic system requires a significant revision of cell culture protocols. Precisely, multiple factors distinguish microfluidic from macroscopic cell cultures, such as different culture surfaces, reduced media volumes, and vastly different rates of, and methods for, medium exchange [21]. Additional challenges include the ability to reproduce the architectural complexity of biological tissues and organs *in vitro* in a miniaturized system, as well as methods to connect these individual structures for recapitulating tissue/organ interactions. Because the development of OAC is labor and time intensive, microfluidic chips are currently mainly used as a research tool, while patient-derived organoids can be cultured more directly for precision medicine purposes. Ultimately, organoid-derived epithelial cells reflecting patients’ specificity integrated into OAC will lay the foundation for future applications in personalized therapy.

## A PROMISING PRECLINICAL MODEL FOR DRUG DEVELOPMENT

Currently, 9 out of 10 experimental drugs progressing from discovery toward development fail in clinical studies [22]. Recent reports have put the final price of bringing a drug to the market at approximately $1 billion dollars, with an estimated research time running into multiple years [10]. In Waring *et al.*’s retrospective analysis of drug development [23] including 605 candidates, nonclinical (*i.e.*, preclinical) toxicology was the highest cause of drug attrition, accounting for 40% of failures in Research and Development (R&D) programs. Out of the few candidates that were moved forward to the Development stage, 35% attrition in phase II studies was caused by failure to demonstrate clinical efficacy. These high failure rates highlight the urgent need for alternative screening systems at the early stage of the R&D lifecycle. Stem cell-derived mini-guts constitute an excellent model to identify new molecular pathways that could lead to novel therapeutic approaches. The potential of 3D organoids to better reflect the biology of the *in vivo* intestinal epithelium makes it a physiologically relevant platform for high-throughput screening of drug candidates.

Yet, the success of therapeutic approaches based on stem cells requires an improvement of disease models to more faithfully recapitulate human phenotypes, including the use of animals that have organs comparable in size and physiology to those of humans [24, 25]. While rodents represent an important model for dissecting mechanisms of many human diseases, there is growing concern about the limitations of these animal models with regards to recapitulation of disease pathogenesis in humans. This was recently exemplified by the failures of the anti-IL17/IL13/IL10 candidate drugs in IBD clinical trials. In fact, the need for large animal models to improve translational science has been widely emphasized by It the National Institute of Health [26, 27]. As of today, porcine organoids are by far the most popular large animal model used for biomedical GI research [28–31]. There are however several important limitations of the porcine model. These include: (i) the use of induced/artificial models of inflammation to reproduce part of the pathophysiology of the disease; (ii) the absence of non-invasive medical techniques, such as endoscopy, for long-term follow-up studies; and (iii) the cost associated with these studies, since pigs are typically culled after each experiment.

Of the large animal species used in translational GI research, the dog is especially relevant because canine gut physiology, diet, and intestinal microbiota are considered to be highly comparable to that of humans [32–39]. Interestingly, studies focusing on the relationship between the composition of the gut microbiota and the development of intestinal diseases have shown striking similarities between dogs and humans, but diverging results between mice and humans [28, 40]. Importantly and similar to humans, dogs spontaneously develop severe intestinal diseases such as IBD and CRC [36, 41–44] (Table I). Therefore, dogs can fulfill a pivotal role as a clinically relevant animal model in the translation from mouse to man. In contrast to pigs and mice, the natural occurrence of chronic enteropathies in dogs allows *in vivo* clinical trials to be conducted, in which the efficacy and safety of drug intervention can be monitored longitudinally, similar to clinical studies in humans. Long-term, the complementary information generated in *ex vivo* organoids and through canine *in vivo* clinical studies can be used to select the most efficacious and safe therapeutics for clinical testing in human patients.

## CLINICAL APPLICATIONS: PRECISION AND REGENERATIVE MEDICINE

### For Personalized Oncology

Undoubtedly the greatest promise of the organoid model lies in its potential application to precision medicine. The ability to grow organoids from patients with intestinal cancer enables personalized testing of a wide range of therapeutics (and combination therapies) within weeks. CRC is one of the most prevalent and debilitating disorders of the GI tract, causing more than half a million deaths annually worldwide [45]. The disease develops through at least three major pathways, including (i) chromosomal instability, (ii) mismatch repair, or (iii) CpG island methylator phenotype (CIMP) [46, 47]. The absolute number and combination of genetic alterations in CRC confounds our ability to determine the contribution of each of these potential oncogenes on tumor development. The organoid model is perfectly suited to study the effect of genetic and epigenetic alterations on cellular differentiation and proliferation. Specifically, Calvin Kuo *et al.* from the University of Stanford have shown the utility of the highly tractable organoid system for modeling the biology of CRC [48]. There is tremendous potential for using organoids to develop more personalized medicine approaches, *i.e.*, being able to treat each individual patient with drugs that are more likely to be efficacious and safe [49, 50]. Using surgically resected tumors from 20 previously untreated CRC patients, van de Wetering *et al.* [51] developed automatized drug sensitivity platform in 3D organoid culture and correlated chemotherapeutic drug sensitivity with genomic profiles to identify molecular signatures associated with drug responses. From their analyses, a single colonoid culture was particularly sensitive to Wnt secretion (porcupine) inhibitors while carrying a gene mutation in the negative Wnt feedback regulator RNF43. These authors further confirmed the activity of cetuximab in a subset of KRAS wild-type organoids reflecting observations made in the clinic [52] and demonstrated Nutlin-3a effectiveness in TP53 wild-type organoids. Similarly, organoids from primary human pancreatic ductal adenocarcinoma have been expanded for drug screening in human patients showing poor response to chemotherapy protocols [53].

Various imaging techniques such as ^18^F-fluorodeoxyglucose (FDG)-PET have been previously explored as a predictor of stem cell response, but they usually lack the resolution and sensitivity to accurately quantify therapeutic response on a cellular level [54]. Walsh *et al.* [55] showed that optical metabolic imaging (OMI) of organoids derived from primary tumors can predict therapeutic response of anticancer drug responses *in vivo*. Their results indicate that OMI is sensitive to therapeutic intervention as early as 24 h after treatment of organoid with candidate drugs while resolving cell sub-populations with distinct metabolic phenotypes. Although most of the laboratory protocols for primary tumor organoids require the use of fresh tissues (which limits their clinical use), the same authors recently showed that viable organoids could be grown from bulk tissues slowly frozen in DMSO-supplemented media [56]. They further demonstrated that the drug response of organoids from frozen samples correlated well with that of organoids obtained from fresh biopsies such that organoids can be collected, frozen, and used subsequently for drug testing purposes.

### To Treat Patient-Specific Congenital Defects

Diseases that are associated with whole organ intestinal failure manifest with severely reduced function of the intestinal epithelial layer. As such, a growing number of congenital diseases of the gut results in generalized loss of nutrient absorption capacity in newborns. In addition, extensive bowel resection in IBD or necrotizing enterocolitis can develop into functional short bowel syndrome. These conditions often result in severe malnutrition and a dependence on intravenous parenteral nutrition. Using a dextran sulfate sodium (DSS)-induced model of IBD, Yui *et al.* [57] demonstrated the feasibility of colonic organoid transplantation in mice. Transplanted cells adhered to and covered superficially damaged tissues in only a few weeks. One month after transplantation of colonic organoids, donor-derived cells were able to form self-renewing intestinal crypts that appeared to be functionally and histologically normal. Similar observations were made in a subsequent study by Fordham *et al.* [58] following transplantation of immature intestinal progenitor cells in DSS-treated mice.

Additionally, orthotopic transplantation of *engineered* **organoids** has the potential to repair or replace damaged epithelial tissues associated with chronic GI disorders, such as IBD, tuft cell enteropathy, cobalamin deficiency, and cystic fibrosis (CF). The proof-of-principle use of genome editing in organoids was demonstrated in a study by Schwank *et al.* [59] in two CF patients. In this study, CRISPR/Cas9 techniques were used to correct the anomalous CF transmembrane conductance regulator locus by homologous recombination in cultured intestinal stem cells. Following genome editing, the corrected allele was expressed and demonstrated to be fully functional in clonally expanded organoids. A similar approach was used in COMMD1-deficient dogs where gene supplemented hepatic organoids facilitated restoration of liver function as an effective strategy to treat copper storage disease. COMMD1 deficiency is an autosomal recessive genetic disorder which predisposes dogs to accumulate copper in hepatic cells. As a result, these dogs develop copper-induced hepatitis which is clinically very similar to Wilson’s disease in humans [60, 61]. Functional assays to determine intracellular copper accumulation have been performed using a fluorescence-based copper sensor [62–65]. This assay showed that *COMMD1*^−/−^ hepatic organoids had a higher intracellular accumulation of copper compared to normal organoids. Yet, after *ex vivo* gene correction in hepatic organoids from dogs with COMMD1 deficiency using lentivirus transfection, the normal phenotype was restored and hepatic organoids could be transplanted into canine patients. This study therefore serves as a proof-of-concept that gene therapy in hepatic organoids of dogs carrying genetic mutations is possible and can, in the future, be developed into regenerative medicine applications. Another good example where gene editing into canine organoids could be used as a preclinical model for human congenital disorders is vitamin B12 deficiency (known as Imerslund-Gräsbeck syndrome), which also develops spontaneously in certain dog breeds, such as Border Collies, Giant Schnauzers, and Beagles [66].

### For Precision and Translational Medicine in Dogs

Similar to humans, many GI disorders in dogs such as IBD, CRC, and congenital absorption defects specifically affect the epithelial layer of the intestine. The organoid model provides a unique approach for understanding how gene polymorphisms can influence the response to therapeutic drugs with potential applications to personalized therapy in veterinary medicine.

In fact, given the many similarities in gene polymorphisms between dogs and humans with IBD, there is a significant opportunity for the use of organoids from diseased dogs to identify new molecular targets and therapeutic strategies for human patients. A copious amount of literature has established that human and canine IBD shares common clinical and molecular features [34–39, 67–76]. In agreement with studies in humans, research on IBD in affected dogs has led to the hypothesis that genetic factors and enteric bacteria can play a pivotal role in the pathogenesis of these disorders, owing to the abnormal intestinal response to commensal microflora. More specifically, a recent study showed that several Toll-like receptors (2, 4, and 9) are upregulated in intestinal biopsies of dogs with IBD [64]. These results are consistent with previous descriptions in humans suffering from IBD. lgarashi *et al.* [72] and Kathrani *et al.* [36, 73–75] have shown that, similar to humans, single nucleotide polymorphisms (SNPs) in NOD2 and TLR5 play an important role in canine IBD. Even more recently, Peiravan *et al.* (manuscript under review) have investigated the genetics of IBD in German Shepherd dogs and identified SNPs in several genes (*e.g.*, IL4, IL13, and SLC22A5) that are known to be associated with IBD in humans. Finally, the modulation of intestinal lamina propria lymphocytes P-gp expression seems to play a similar role in both human and dog IBD. In IBD patients scarcely responsive to steroid treatment, P-gp is highly expressed and, in dogs showing a good response to treatment, this protein is in fact modestly represented [66].

The clinical relevance of the canine organoid model to dog and human precision medicine also applies to colon cancer, for which multiple gene mutations commonly found in humans (*e.g.*, tumor suppressors APC and TP53) have been shown to be present in similar frequencies in canine CRC biopsies [42–44]. The development of an *ex vivo* model of canine CRC, representing samples of clinical and molecular diversity similar to that seen in people, would therefore constitute an essential step to promote translational and comparative research on CRC.

## FUTURE DIRECTIONS

Although very promising, various challenges must be overcome before the organoid model can be routinely used in clinical practice. In particular, intestinal organoids typically lack several essential components of the native digestive microenvironment, including cells of the adaptive and innate immunity, as well as the enteric nervous system. In addition, the organoid system does not recapitulate the gradient of nutrients which is present along the cryptvillus axis, or mimic biomechanical forces that stem cells encounter *in vivo* [10]. There is therefore an increasing demand to integrate the organoid system into a microfluidic organ-on-a-chip device, as described by Takebe *et al.* [77] in a paper titled “Synergistic Engineering: Organoids meet Organs-on-a-Chip.”

Currently, most applications of organoids for precision medicine are related to the screening of anticancer therapeutics, with only few descriptions in other indications such as infectious diseases. This is partly due to the difficulty of co-culturing bacteria or viruses with epithelial cells in the organoid system. Specifically, human noroviruses—one of the most common causes of epidemic gastroenteritis over the world—have resisted cultivation efforts until very recently, when Mary Estes’s group showed that these viruses replicate in human organoid systems [78, 79]. This model has since then proven useful in testing anti-viral drugs by measuring viral load in infected organoid monolayers.

There are additional limitations to the use of organoids for regenerative medicine. Organoid cultures usually depend on mouse sarcoma-derived Matrigel, which precludes transplantation of organoids into humans [18]. Future considerations should therefore include alternative growth media compositions and culture protocols to maximize the yield of intestinal stem cells used for regenerative purposes [80]. Yet-to-be-performed experiments must include transplantation studies in large animal models to assess long-term safety, efficacy, and tumorigenicity of organoids for treatment of chronic GI diseases. Additional concerns include the method of tissue collection, the optimal route of mucosal delivery (presumably via endoscopy), and design of a suitable delivery vehicle to protect and sustain cells during transit while allowing for mucosal adhesion [81].

Demonstration of drug efficacy and safety in animals remains the best way to gain sufficient experience to initiate ethically designed human trials. Given the many limitations of the mouse model, there is a critical need to develop well-characterized organoid systems in more relevant animal disease models.

**Fig. 2:**
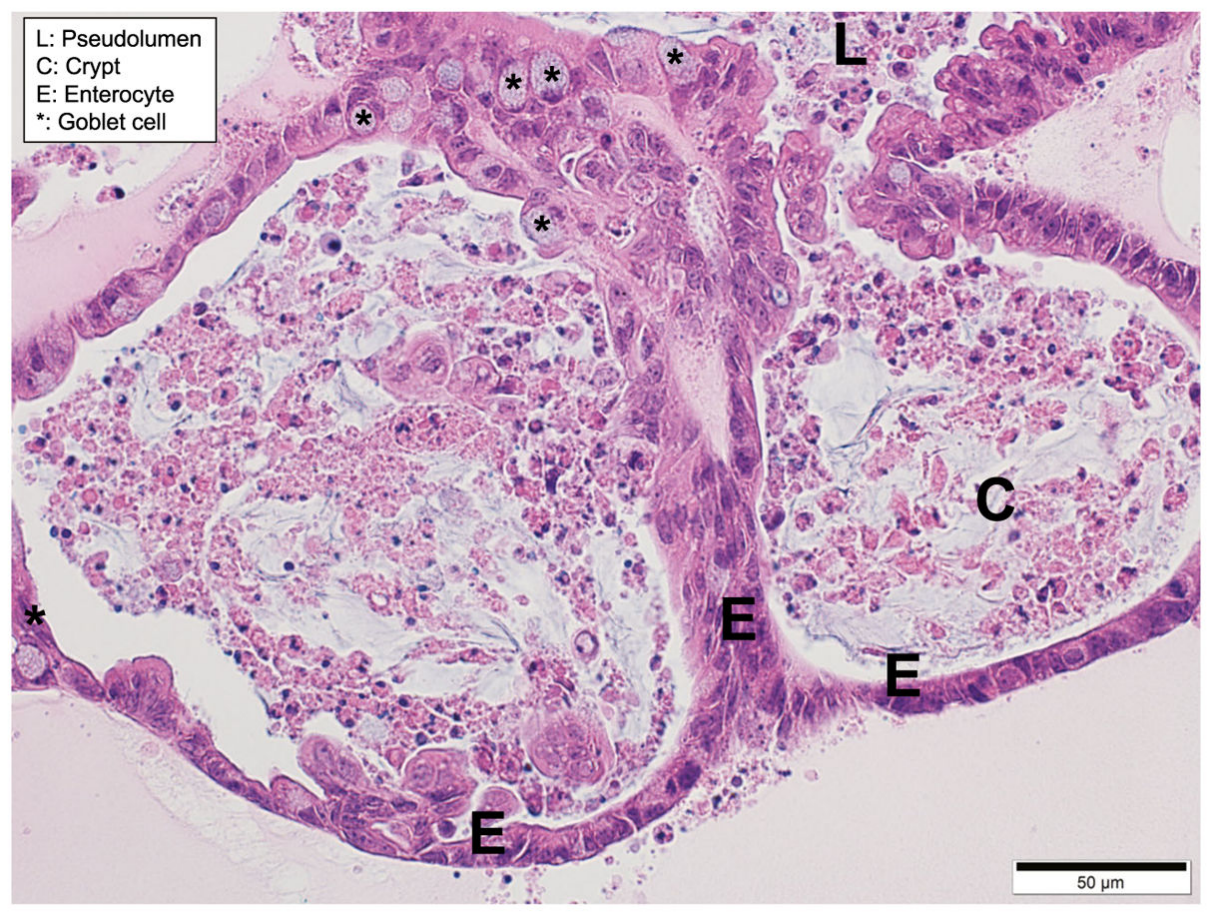
As the canine organoids grow, spheroids develop into large complex structures with a pseudolumen (L) and crypt-like (C) projections, similar to human organoids. Canine small organoid, 5-um-thick, paraffin-embedded section stained with H/E at ×400

Using published protocols for mouse, pig, and human organoid culture [82–84], our laboratory is currently developing canine organoids in an effort to find cures for humans and dogs suffering from similar diseases like IBD and CRC. Our preliminary data, which include live cell bright-field imaging, Trypan Blue viability staining, and hematoxylin and eosin (H/E, Fig. 2) showed a budding organoid morphology consisting of differentiated columnar epithelium, and spheroid formation in a high Wnt conditioned medium, similar to that seen in human organoids. Further characterization of the canine organoid system, including transmission electron microscopy, immunohistochemistry, RNA *in situ* hybridization, and gene expression analysis is underway. This will be an important step toward the validation of canine organoids as a relevant *in vitro* system for modeling human diseases.

## CONCLUDING REMARKS

Organoids are a powerful mechanistic tool for identifying molecular targets that are relevant to the pathogenesis of chronic intestinal diseases. They can also be used as a platform for the screening of drug candidates that target the epithelial components of diseases such as IBD and CRC. However, until it has been confirmed that organoids can express patient-specific disease pathways, its main utility is to support potential therapeutic options and drug discovery as an early exploratory tool. The hope is that this Commentary will stimulate support for the research needed to enable organoids to serve as a tool guiding medical decisions within the clinical setting.

In addition, the possibility to grow organoids representative of the main targets for GI drug-related toxicity (*e.g.*, gut, liver) opens up new avenues for complementary animal-based toxicology studies. Importantly, because organoids are derived from individuals with different genotypes, environmental risk factors, and drug sensitivity profiles, they are a highly relevant model system for targeted and personalized therapy. Combination of organoids with microfluidic chips for the integration of patient-derived cells could lay the foundation for the future of precision medicine. Ultimately, orthotopic transplantation of organoids has the potential to repair or replace damaged epithelial tissues associated with a wide range of intestinal disorders, including IBD, CF, and tuft cell enteropathy.

Yet, the success of therapeutic approaches based on organoids requires a substantial improvement of the animal disease models they are derived from. Specifically, there are growing concerns about the ability of murine models to faithfully recapitulate human phenotypes. This is consistent with several NIH initiatives (*e.g.*, *PAR*-16-094 and PAR-16-322) aiming at improving existing animal models to support stem cell research and the development of new therapeutic discoveries for digestive diseases. The dog is a particularly relevant species since it shares similar environmental, genomic, anatomical, and intestinal physiologic features with humans. Our preliminary data show that canine organoids can be successfully grown from intestinal crypts, and maintained in culture long-term. This is a significant step toward the development of a completely new animal model system for GI research, with the added benefit that dogs, similar to humans, develop naturally occurring enteropathies. By integrating this complement of knowledge, organoids can one day serve as a tool to inform clinicians regarding preferred patient-specific therapeutic options both in veterinary and human medicine.

## Figures and Tables

**Table I:** Comparative Features of IBD and Colorectal Cancer in Different Mammalian Species

Feature	Human	Dog	Rodent
Genetic basis	Yes	Yes	Engineered
Etiology	Multifactorial	Multifactorial	+/− Multifactorial
Intact immune system	Yes	Yes	+/−
Gut microbiota role	Yes	Yes	Yes
Blood in stool	Yes	Yes	Yes
Diarrhea	Yes	Yes	Yes
Definitive diagnosis	GI mucosal biopsy	GI mucosal biopsy	GI mucosal biopsy
Longitudinal studies	Yes—endoscopy + histology	Yes—endoscopy + histology	No
Cancer treatment	Surgery + chemotherapy	Surgery + chemotherapy	N/A
IBD treatment	Diet + drugs	Diet + drugs	Drugs
Disease heterogeneity	Yes	Yes	Variable
